# Highly effective microporous and mesoporous metal–organic frameworks for effective ivermectin adsorption in water treatment and delivery systems†

**DOI:** 10.1039/d5ra01662b

**Published:** 2025-04-30

**Authors:** Ola Gamal, Walaa A. Moselhy, Mohamed Taha

**Affiliations:** a Environmental Science and Industrial Development Department, Faculty of Postgraduate Studies for Advanced Sciences (PSAS), Beni-Suef University Beni-Suef Egypt; b Toxicology and Forensic Medicine Department, Faculty of Veterinary Medicine, Beni-Suef University Beni-Suef 62511 Egypt; c Materials Science and Nanotechnology Department, Faculty of Postgraduate Studies for Advanced Sciences (PSAS), Beni-Suef University Beni-Suef Egypt mtaha@psas.bsu.edu.eg

## Abstract

Metal–organic frameworks (MOFs) are an emerging class of materials with exceptional porosity and tunable structures, making them highly effective for adsorbing harmful impurities from water. These properties render MOFs particularly suitable for environmental remediation. However, evaluating all available MOFs is impractical due to their vast number. To address this, we employed computational screening using Grand Canonical Monte Carlo (GCMC) simulations on a database of over 14 000 MOFs to identify the most promising candidates for antiparasitic drug (ivermectin, IVM) adsorption, drug delivery, and membrane filtration. The GCMC simulations identified 584 MOFs with potential applications. Among them, 147 MOFs demonstrated strong IVM adsorption capabilities, making them suitable for drug delivery and adsorption applications. The remaining 437 MOFs exhibited properties ideal for membrane filtration, specifically for reverse osmosis and nanofiltration to separate IVM. The loading capacity and isosteric heat of the 147 MOFs at 101.325 kPa and 298 K were calculated and correlated with various structural properties, including largest void diameter, pore-limiting diameter, accessible volume, density, and helium void fraction. Molecular dynamics simulations were performed on the most promising MOFs to understand the IVM loading mechanism.

## Abstract 2

This study uses high-throughput screening of 14 000+ MOFs to identify top candidates for ivermectin adsorption. MD simulations reveal key mechanisms, supporting MOF selection for water treatment and drug delivery applications.

## Graphical abstract

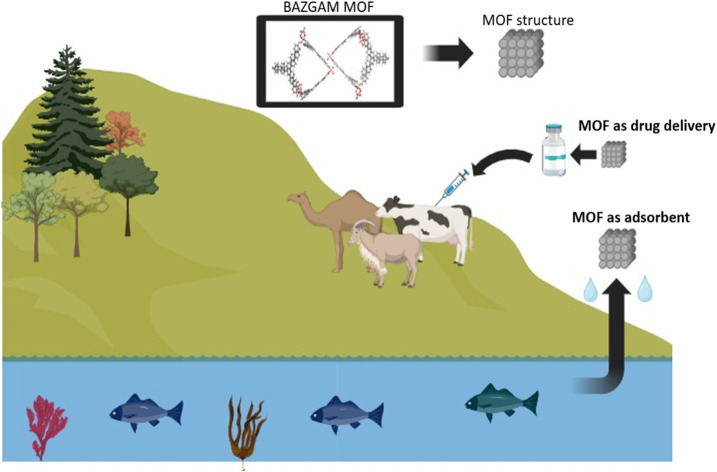

## 1. Introduction

Water availability and quality are essential for sustaining health, biodiversity, and the environment. For instance, clean, potable water is crucial for preventing diseases, supporting agricultural production, and maintaining the vitality of aquatic ecosystems.^1,2^ The environment and water supplies have become contaminated because of the rapid growth of population, lifestyle changes, the construction of new industrial facilities, and rising energy utilisation. One of the most significant environmental problems is the scarcity of water resources and their contamination. When substances are introduced into water, they can alter its physical, chemical, and microbiological properties.^3^ Serious contamination problems can occur if wastewater is released into water streams before treatment.^4^ The contaminants can be categorized as nuclear, chemical, and biological.^3^ Chemical pollutants include substances such as human and animal drugs (*e.g.*, antibiotics and antiparasitics), hormones, plasticizers, and insecticides.^5^

Ivermectin (IVM) is a widely used veterinary antiparasitic medication that is effective against a broad range of parasites. It is used for treating nematodes, ticks, warble flies, lice, and mites in sheep, cattle, horses, and pigs.^6^ IVM is a macrocyclic lactone, which is a branch of avermectins, that is made up of two basic homologue compounds (80% 22, 23-dihydroavermectin B1a and 20% 22, 23-dihydroavermectin B1b). IVM can directly pollute soil through animal waste and contaminate surface and groundwater through discharge from fields. Due to its effectiveness against parasites, IVM has been widely used in the aquaculture industry. After a single oral dose, IVM spread rapidly throughout the water system within a day and accumulated massively in organisms, leaving persistent residues.^7^ In marine water sediment, it had a half-life of over 100 days.^8^ It has been reported that IVM has the potential to directly or indirectly cause genetic damage, which could enable cancer cells to grow and spread.^9^ The study found that IVM may contribute to the degradation of genetic material, leading to genetic instability. IVM has demonstrated genotoxic effects,^9^ as evidenced by various forms of chromosomal abnormalities in somatic and embryonic cells of exposed species, including deletions, fragments, polyploidy, micronuclei formation, and circular chromosome formation. IVM can cause several undesirable side effects in mammals, such as headaches, nausea, diarrhoea, allergic reactions, and stomach pain.^9^ Therefore, it is crucial to remove IVM from water using modern and effective methods.

Despite numerous materials and technologies developed for water decontamination, effectively cleaning sewage remains a significant challenge. Sewage treatment utilizes a variety of methods to eliminate the contaminants, such as adsorption,^10^ separation,^11^ membrane filtration,^12^ photocatalytic degradation.^13^ Adsorption is a phenomenon that occurs on surfaces and is occasionally caused by chemical forces as well as physical ones. Adsorbent refers to a solid surface, and adsorbate refers to a molecule that attaches to it.^14^ Adsorption is an effective method for removing various contaminants from wastewater due to its simplicity, versatility, high efficiency, lack of waste generation, low cost, and the recyclability of adsorbents. Many factors specify the adsorption capacity of the adsorbent, such as the pore structure, surface chemistry, functional groups, and polarity of the adsorbent and adsorbate, as well as the adsorbate's molecular diameter.^15^ Several adsorbents have been investigated for IVM removal from water, exhibiting varying adsorption capacities. Kaolinite-based composites, such as kaolinite-pine cone (115.8 μg g^−1^) and kaolinite-papaya (105.3 μg g^−1^).^16^ Commercial bentonite-based organophilic clay exhibited a capacity of 0.00178 to 0.00388 g g^−1^,^17^ while commercial charcoal demonstrated a higher efficiency of 173 to 203 mg g^−1^.^18^ A chitosan-based magnetic adsorbent reported an adsorption capacity of 81.86 mg g^−1^.^19^ Polyamidoamine functionalized graphene oxide-SBA-15 mesoporous composite exhibited a capacity of 291.8 μg g^−1^.^20^

Metal–organic frameworks (MOFs) have proven to be highly efficient adsorbents for removing a variety of harmful pollutants due to their high porosity, customizable pore structure, and ease of functionalization.^21^ MOFs were first introduced by Omar Yaghi *et al.* as a novel class of materials that form through the self-assembly of organic linkers and metal ions or clusters.^22^ Its performance surpasses that of commonly used materials like zeolite and clay.^23^ The pore size and shape of MOFs can be tuned by modifying the ligands and metal clusters to achieve specific functions.^24^ The unique properties of MOFs position them as promising materials for removing a wide range of contaminants including textile dyes, pesticides, drugs, paints, heavy metal ions, and organic solvents.^25–30^ MOFs also can be used as a drug delivery system to deliver drugs in a controlled manner, thereby increasing effectiveness and reducing side effects.^31,32^ MOFs possess an exceptionally large surface area and functionally tunable pores that can be tailored to accommodate specific guest molecules. This unique characteristic allows for efficient interaction between the guest molecules and the MOFs, facilitating a controlled and gradual release of the encapsulated substances.^33^ One of the significant advantages of MOFs is their biodegradability, and ability to decompose after a reasonable period of stability during delivery. This decomposition prevents the accumulation of MOFs in the environment, minimizing potential ecological risks associated with persistent materials.^34^

As of recent estimates, there are over 100 000 MOFs that have been experimentally synthesized and characterized.^35^ This number is constantly growing as researchers continue to synthesize and explore new MOF structures for various applications. The vast diversity of MOFs is due to the countless combinations of metal nodes and organic linkers that can be used to create these materials. In 2014 Chung *et al.*, conducted research on Computation-Ready, Experimental Metal–Organic Frameworks, constructing more than 4000 computation-ready MOFs of porous structures. In 2014, Chung *et al.* constructed a database of over 4000 computation-ready, experimental MOFs (CoRE MOFs), which provided a valuable resource for researchers.^36^ The structures of these MOFs were taken from the Cambridge Structural Database (CSD). This database provides a foundation for computational studies of MOFs, such as simulations of gas adsorption, catalysis, and drug delivery. These simulations can help researchers understand the properties and behavior of MOFs in different applications. In 2019, Chung *et al.* updated their CoRE MOF database to include over 14 000 porous, three-dimensional MOF structures.^37^ This update was sourced from the CSD and a Web of Science search. Grand Canonical Monte Carlo (GCMC) simulations have been extensively employed in MOF screening for gas separation and adsorption applications, such as CO_2_ capture, CH_4_ storage, H_2_ storage, and water harvesting.^38–41^

In this study, we employed a computational screening approach to evaluate the potential of 14 000 MOF structures for IVM adsorption using GCMC simulation. The initial screening process selected MOFs with a largest cavity diameter (LCD) greater than 12 Å, corresponding to a pore-limiting diameter (PLD) of 2.67 Å, given that the molecular dimensions of IVM are approximately 11.2 × 22.0 Å. This filtering process identified 584 MOFs with LCDs ranging from 12 Å to 71 Å. Generally, MOFs with an LCD below 20 Å are classified as microporous, while those with an LCD of 20 Å or greater are considered mesoporous. These selected MOFs are expected to exhibit the highest capacity for IVM adsorption within their pores. To further investigate adsorption mechanisms, molecular dynamics (MD) simulations were performed on the top-performing MOFs, analyzing adsorption sites and interaction forces. Given the rapid increase in MOF production, experimental evaluation of all potential candidates is impractical. Therefore, computational screening has become a reliable and efficient approach to identify the most promising MOF candidates, providing valuable insights for experimental researchers.

By providing a ranking based on expected performance, our approach eliminates the need for random MOF selection, which often results in choosing materials with inadequate pore sizes for the target adsorbate. Such limitations restrict adsorption to the MOF surface rather than allowing penetration into internal pores, thereby reducing overall capacity and efficiency. Surface adsorption alone significantly limits the adsorption potential of the material. Previous studies that combined experimental and computational approaches relied on random MOF selection,^42^ leading to cases where the adsorbate remained confined to the surface without accessing the internal pores. This outcome contradicts the fundamental advantage of MOFs, which is their high porosity and tunable structure designed to maximize adsorption efficiency.

## 2. Computational methods

The crystallographic information files (CIF) were taken from the CoRE MOFs 2019 database. All simulations were carried out using BIOVIA material studio program (https://www.3ds.com/products/biovia/materials-studio).^43^ The Universal Force Field (UFF)^44^ was employed throughout all stages of the study, including geometry optimization, MC, and MD simulations, to evaluate the energies of the MOF frameworks, IVM, and water molecules. UFF provides a comprehensive and transferable parameter set based on atomic connectivity and elemental properties, making it well-suited for modeling systems with diverse coordination environments, such as MOFs with a wide range of metal centers and organic linkers. Atomic charges for all atoms in the system, including those in the MOFs, IVM, and water, were calculated using the Charge Equilibration (QEq) method,^45^ a fast and widely used empirical approach that estimates partial atomic charges by equilibrating electronegativity and atomic hardness across the system. While we acknowledge that this method may not capture all aspects of charge distribution with the same precision as quantum mechanically derived charges, it enables efficient treatment of large and chemically diverse databases in high-throughput computational workflows. Although experimental data for IVM loading in MOFs is currently unavailable, limiting direct validation of the simulation results, both UFF and QEq have been widely and successfully used in previous MOF-related adsorption studies, with outcomes that align well with experiment1al findings [*e.g.*, ^46,47^]. Ashraf *et al.*^46^ employed the UFF forcefield and QEq charges to model MOF–organic pollutants (*e.g.*, ZIF-8, ZIF-67, FeTCPP, CuTCPP, and ZnTCPP with methomyl, ethion, prothiofos, and diazinon), with simulated loadings closely matching experimental values. Similarly, simulations of ibuprofen loading in MIL-53(Fe), MIL-101(Cr), MOF-74, CD-MOF-1, and bio-MOFs-1, −11, and −100 using these simulation parameters also showed good agreement with experimental uptake data.^47^ Therefore, the methodology applied here is appropriate for the scope of a high-throughput screening study and provides valuable qualitative and semi-quantitative insights into IVM adsorption behavior across a large set of MOF materials.

IVM and water molecules were first geometry-optimized using the Forcite module to ensure energetically favorable conformations prior to sorption simulations. Both optimized molecules were treated as sorbate species in the GCMC simulation to mimic competitive adsorption under aqueous conditions. The simulation temperature was set to 298 K and the pressure to 101.325 kPa, corresponding to standard ambient conditions under which water exists in the liquid state. Although the explicit mole fractions of IVM and water were not predefined, the GCMC approach allows molecules to enter and exit the simulation cell freely, equilibrating the system with a virtual reservoir at constant chemical potential. The simulations were carried out using the Sorption module in BIOVIA Materials Studio, which enables the simultaneous adsorption of multiple components under fixed thermodynamic conditions. The Metropolis sampling technique was used in the GCMC simulation with the following steps: exchange (39%), translation (20%), conformer rotation (20%), rotation (20%) and regrowth (2%). In general, GCMC simulations take 1–5 ×10^6^ MC steps to equilibrate, and then they take a comparable amount of time to gather data.^48^ The simulation was designed in two stages to strike a balance between computational efficiency and reaching equilibrium. A stage of equilibration: (3 × 10^6^) steps and a production: (3 × 10^6^) steps to gather data. Cubic spline truncation method combined with atom-based summation method with buffer width 0.5 A, cutoff distance 12.5 Å applied to compute van der Waals. To calculate the electrostatic forces, we used Ewald summation method. The dimensions of the simulation box were selected so that they were at least double the cutoff distance in each direction.

To clarify the absorption mechanisms of the top three candidate MOF-IVM-water systems, the initial configurations for the MD simulations that followed were based on the lowest-energy configurations from the MC simulations. This confirmed that the systems were equilibrated before starting the MD simulations. The parameters used in MD simulation were initial velocity (random), ensemble (NVT), temperature (298 K), time step (1 fs), frame output every (500 steps), total simulation time (1000 ps), number of steps (1 000 000), and thermostat (Nose).

## 3. Results and discussion

### 3.1. MC simulation

**Scheme 1:**
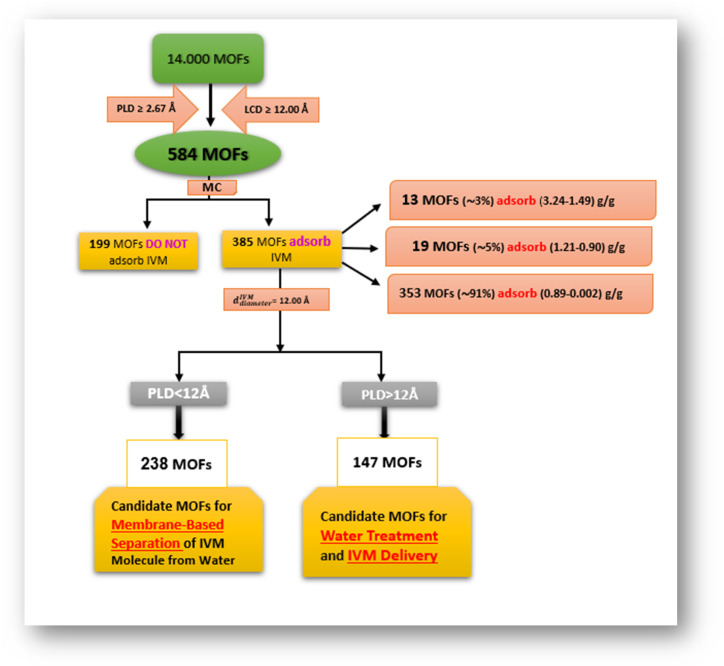
Screening of 14,000 MOFs for IVM adsorption and separation.

The LCD is known as the largest sphere that can fit inside the MOFs' pore.^49^ If the size of the IVM molecule is larger than the LCD, it cannot penetrate or be absorbed by the MOF. By evaluating the size limit of the IVM molecule (22.04 Å × 11.56 Å) and performing MC simulations on 584 MOFs from the (ASR) CoRE MOF 2019 database, 385 MOFs were identified as potential adsorbents for IVM, while 199 MOFs were classified as non-adsorbents (Scheme 1). Among the adsorbents, a wide range of capacities was observed: 13 MOFs (approximately 3%) exhibited high adsorption (1.49–3.24 g g^−1^), 19 MOFs (approximately 5%) showed medium adsorption (0.90–1.21 g g^−1^), and the majority, 353 MOFs (approximately 91%), displayed lower adsorption (0.002–0.89 g g^−1^). The PLD of a MOF acts as a molecular filter, controlling the size of molecules that can access its internal surface based on their size.^49,50^ This property is crucial for MOF selectivity in the process of separation^51^ and effectiveness in various applications. A larger PLD allows for a greater storage capacity, as it enables larger inner cavities within the MOF, accommodating more absorbed molecules. If the size of the adsorbed molecule exceeds the PLD, even with a large LCD, adsorption is hindered because the molecule cannot access or interact with the internal parts of the MOF. Conversely, if the molecule is smaller than the PLD, diffusion and release occur more readily. However, when the PLD closely matches the size of the target molecule, selectivity can be enhanced.

Using MD simulation to model a single IVM molecule in 1000 water molecules at 298 K and 1 bar, the IVM molecule (*d*^IVM^_diameter_) was found to have a kinetic diameter of approximately 12.00 Å in water. Out of the 385 MOFs capable of adsorbing IVM, 238 act as barriers because their PLDs are less than 12.00 Å, preventing the IVM from accessing their internal sites. These 238 MOFs have PLDs that selectively allow the passage of water molecules while blocking IVM molecules, making them suitable for membrane separation applications involving IVM. The remaining 147 MOFs, with PLDs greater than 12.00 Å, permit IVM molecules to enter their internal sites. These MOFs are excellent candidates for water treatment applications, as their large LCD and PLD values enable efficient IVM adsorption.

Among them, approximately 13 MOFs (∼9%) exhibit high adsorption capacities ranging from 3.24 to 1.49 g g^−1^, while 20 MOFs (∼14%) have medium adsorption capacities between 1.21 and 0.884 g g^−1^. The remaining 114 MOFs (∼77%) show lower adsorption capacities ranging from 0.882 to 0.04 g g^−1^ (see Scheme 1 and ESI†).

**Fig. 1:**
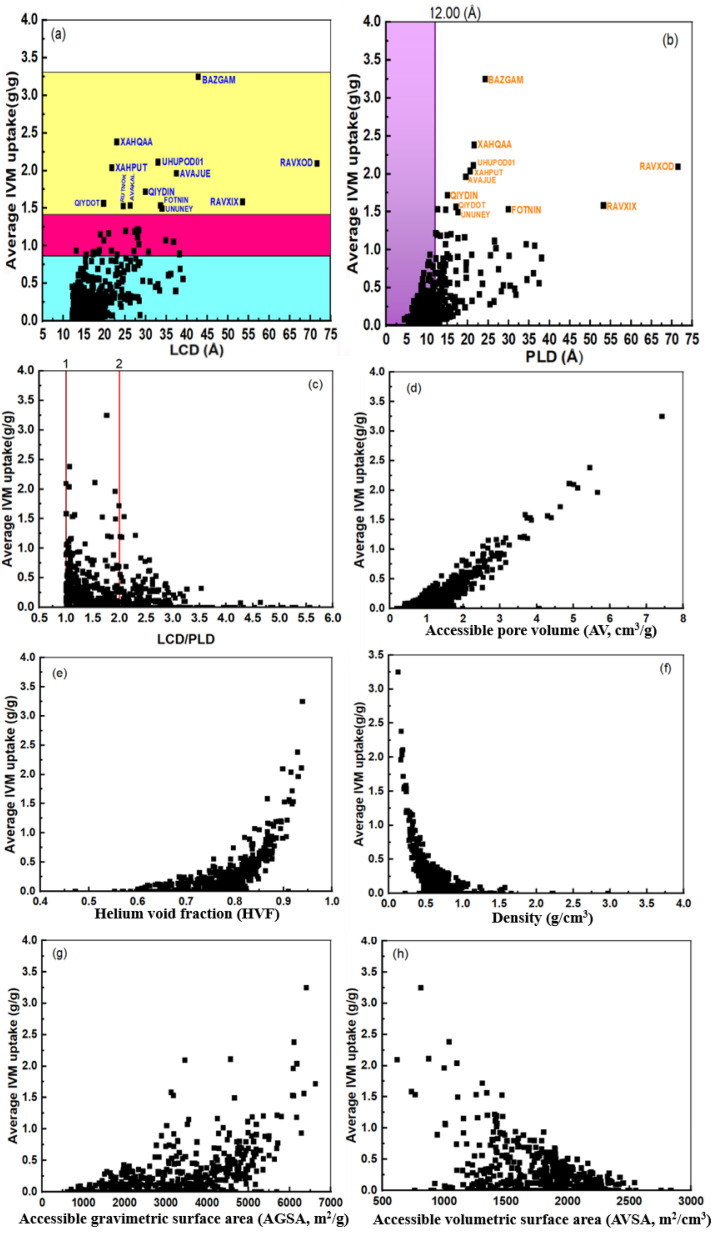
The relationship between loading capacity of IVM and (a) LCD, (b) PLD, (c) LCD/PLD, (d) AV, (e) HVF, (f) density, (g) AGSA and (h) AVSA.

Fig. 1 shows the correlations between IVM uptake and various MOF structural descriptors, such as LCD, PLD, LCD/PLD ratio, accessible pore volume (AV, cm^3^ g^−1^), density (g cm^−3^), helium void fraction (HVF), accessible volumetric surface area (AVSA, m^2^ cm^−3^), and accessible gravimetric surface area (AGSA, m^2^ g^−1^). Fig. 1a displays a weak correlation between IVM uptake and LCD, suggesting that LCD alone is insufficient for accurately predicting IVM adsorption. The sample data ranges from 12 to 43 Å, with two outliers MOFs at 53 Å and 71 Å, corresponding to RAVXIX and RAVXOD, respectively. A similar trend was observed in the correlation between IVM uptake and PLD, further emphasizing that individual structural descriptors like LCD and PLD have limited predictive power on their own (Fig. 1b).

By calculating the ratio between LCD and PLD (LCD/PLD)^51^, we can roughly characterize the pore morphology within the MOFs,^50^ providing insights into their adsorption and release capabilities. When the LCD/PLD ratio equals 1, it implies that the diameter of the largest cavities within the framework is equal to the narrowest areas in the percolating channels. This means that the structure has uniform pore sizes, with no significant bottlenecks or variations in pore diameter. A ratio greater than 1 indicates that the structure resembles a network of large cavities connected by narrow channels or windows. This scenario creates “bottleneck” pores, where the narrow channels limit access to the larger cavities. Such structures can exhibit complex adsorption behavior due to the restricted diffusion through these narrow channels.^52^ Conversely, if the ratio is less than 1, the channels are wider than the cavities, suggesting a more open and accessible pore structure. Fig. 1c shows that IVM uptake increases as the LCD approaches or doubles the PLD, but decreases at higher LCD/PLD ratios.

The accessible pore volume AV is known as the volume space that the center of an adsorbate molecule can access.^53^ AV is one crucial feature of MOFs because it affects how molecules can be transported inside the structure. The pore volume may be represented by the accessible volume through the MOF's center of mass.^54^Fig. 1d shows that IVM uptake increases with larger AV, indicating more potential for IVM molecule storage in MOFs with higher accessible pore volumes. This suggests that maximizing AV can enhance the adsorption capacity of MOFs.

The void fraction, often referred to as porosity, is a measure of the empty or void spaces within a material, expressed as a fraction between 0 and 1, where 0 represents a completely occupied volume and 1 represents a completely empty volume.^55^ Helium gas, due to its inertness, is commonly used to measure the void fraction, which is referred to as the helium void fraction (HVF). Fig. 1e shows that IVM uptake remains relatively stable with a slight increase until the HVF reaches approximately 0.8. Beyond this point, IVM uptake increases sharply. This observed threshold suggests that there is an optimal range of porosity for maximizing IVM uptake. This could be due to the increased accessibility of adsorption sites at higher porosities, allowing more efficient diffusion and adsorption of IVM molecules. Fig. 1f shows a reverse relationship between IVM uptake and density. High IVM uptake corresponds to less empty space in the MOF, which is associated with higher density.

Fig. 1g and h shows the relationship between the IVM uptake and the accessible gravimetric surface area (AGSA, m^2^ g^−1^) and the accessible volumetric surface area (AVSA, m^2^ cm^−3^) of MOFs, which are respectively related to the MOF mass and volume as well as the total surface area open to contact with the adsorbate. In Fig. 1g the uptake of IVM increased slightly with the increase of AGSA up to 4500 m^2^ g^−1^, after this value the IVM increases sharply except for higher IVM uptake (0.5 to 2.0) g g^−1^ certain MOFs at (3000), (3500) m^2^ g^−1^. In Fig. 1h the IVM uptake decreases with increasing AVSA until it becomes 0 when the AVSA value is 3000 m^2^ cm^−3^.

Table 1 lists 31 MOFs according to their IVM loading capacity, which is in the range of (0.906–3.248) g g^−1^. These 31 MOFs are divided into two groups: the first group of the high-loading uptake contains 15 MOFs in the range of (1.205–3.248) g g^−1^ and the second group of the medium-loading uptake contains 16 MOFs in the range of (0.906–1.197) g g^−1^. These top MOFs exhibit significantly higher IVM loading capacities compared to traditional adsorbents, such as commercial charcoal, which shows markedly lower capacities (0.173–0.203 g g^−1^).^18^ Among the absorbing-MOFs with PLD > 12.00 Å, 117 were found to have uptake values exceeding 0.203 g g^−1^. These results highlight the superior adsorption potential of MOFs for IVM removal, largely attributed to their tunable pore structures, high surface areas, and tailored host–guest interactions. While microporous carbons like charcoal are often cost-effective, they generally lack selectivity and tunability. In contrast, MOFs offer customizable pore structures and functional groups, enabling more selective and efficient adsorption of large, hydrophobic molecules like IVM.

**Table 1:** The top 31 MOFs with PLD >12.00 Å based on the IVM uptakes in the range of (0.906–3.2483) g g^−1^

Rank	Ref. code	Uptake (g g^−1^)	LCD	PLD	LCD/PLD	Isosteric heat (kcal mol^−1^)	Metal
**1**	**BAZGAM**	**3.248**	**42.798**	**24.240**	**1.765**	**61.24**	**Cu**
**2**	**XAHQAA**	**2.379**	**23.035**	**21.607**	**1.066**	**66.9**	**Cu**
**3**	**UHUPOD01**	**2.108**	**33.077**	**21.403**	**1.545**	**57.37**	**Zn**
**4**	**RAVXOD**	**2.090**	**71.641**	**71.501**	**1.001**	**52.45**	**Mg**
**5**	**XAHPUT**	**2.036**	**21.828**	**20.585**	**1.060**	**61.02**	**Cu**
**6**	**AVAJUE**	**1.961**	**37.525**	**19.541**	**1.920**	**54.03**	**Cu**
**7**	**QIYDIN**	**1.715**	**29.975**	**15.022**	**1.995**	**69.62**	**Cu**
**8**	**RAVXIX**	**1.582**	**53.576**	**53.264**	**1.005**	**57.53**	**Mg**
**9**	**QIYDOT**	**1.562**	**19.818**	**17.063**	**1.161**	**64.47**	**Cu**
**10**	**FOTNIN**	**1.535**	**33.621**	**29.929**	**1.123**	**59.94**	**Zr**
**11**	**AVAKAL**	**1.532**	**26.252**	**12.575**	**2.088**	**65.31**	**Cu**
**12**	**RUTNOK**	**1.525**	**24.612**	**14.648**	**1.680**	**62.53**	**Zn**
**13**	**UNUNEY**	**1.495**	**34.006**	**17.637**	**1.928**	**55.38**	**U**
**14**	**CUSYAR**	**1.216**	**28.013**	**12.179**	**2.299**	**52.78**	**Zn**
**15**	**HEXVEM**	**1.205**	**28.431**	**15.917**	**1.786**	**76.53**	**Cu**
16	AVAKEP	1.197	25.175	12.493	2.015	61.85	Cu
17	NIBHOW	1.188	27.510	14.884	1.848	74.71	Cu
18	ADATEG	1.184	27.337	13.339	2.049	59.33	Cu
19	RUBDUP	1.165	21.095	19.246	1.096	62	Zn
20	ECOKAJ	1.150	18.997	17.575	1.080	63.11	Zn
21	jacs.6b08724_ja6b08724_si_014	1.113	28.056	26.589	1.055	61.52	Mg
22	RAVXAP	1.074	34.859	34.355	1.014	57.63	Mg
23	ALEJAE	1.072	19.865	14.568	1.363	55.07	In
24	RAVWUI	1.051	36.787	36.433	1.009	64.64	Zn
25	jacs.6b08724_ja6b08724_si_009	1.018	28.389	26.891	1.055	69.03	Co
26	HOHMEX	0.942	18.784	14.894	1.261	60.92	Cu
27	BIGZUO	0.931	21.723	20.987	1.035	54.26	Ag,Cr
28	ja507269n_si_003	0.928	27.209	25.549	1.064	65.61	Fe,Co
29	RAVWIW	0.919	30.700	30.144	1.018	62.46	Mg
30	XAHPON	0.910	17.295	15.332	1.128	63.51	Cu
31	BIHBAX	0.906	18.574	17.556	1.057	46.4	Ag,Cr

The analysis of the 147 MOFs can be approached from three perspectives: IVM uptake, selectivity, and diffusion. Table 1 specifically focuses on MOFs optimized for IVM uptake, without considering selectivity or diffusion. The five MOFs with the highest loading capacities are BAZGAM, XAHQAA, UHUPOD01, RAVXOD, and XAHPUT. The second aspect focuses on MOFs with PLD close to the *d*^IVM^_diameter_. This ensures high selectivity by preventing larger molecules from entering the internal cavities of the MOFs. However, this strategy results in a slower diffusion rate due to the restricted pore size. In contrast, the third aspect involves MOFs with PLDs larger than *d*^IVM^_diameter_, enabling faster diffusion of molecules. However, this comes at the cost of lower selectivity, as the larger PLD allows both IVM and other molecules to access the internal cavities more easily. Among the 15 MOFs in the first group, CUSYAR is the most selective MOF for IVM molecules, as it has the smallest PLD value. It is followed by AVAKAL, RUTNOK, QIYDIN, HEXVEM, QIYDOT, UNUNEY, AVAJUE, XAHPUT, UHUPOD01, XAHQAA, BAZGAM, FOTNIN, RAVXIX, and RAVXOD. On the other hand, the opposite order of those MOFs indicates a higher diffusion rate. CUSYAR and AVAKAL have the largest LCD/PLD ratios, 2.299 and 2.088, respectively. Based on the selectivity analysis of IVM using the 16 MOFs in the second group, AVAKEP emerged as the most selective MOF due to its smallest pore-limiting diameter (PLD) value. The selectivity ranking of the MOFs is as follows: AVAKEP > ADATEG > ALEJAE > NIBHOW > HOHMEX > XAHPON > BIHBAX > ECOKAJ > RUBDUP > BIGZUO > ja507269n_si_003>jacs.6b08724_ja6b08724_si_014 > jacs.6b08724_ja6b08724_si_009 > RAVWIW > RAVXAP > RAVWUI. The inverse order of this ranking reflects the relative diffusion rates of the MOFs, with those at the lower end of the selectivity ranking exhibiting higher diffusion rates. This information is critical for researchers to identify which MOFs are best suited for specific applications, such as adsorption processes or adsorbate delivery systems, depending on the desired balance between selectivity and diffusion efficiency.

**Fig. 2:**
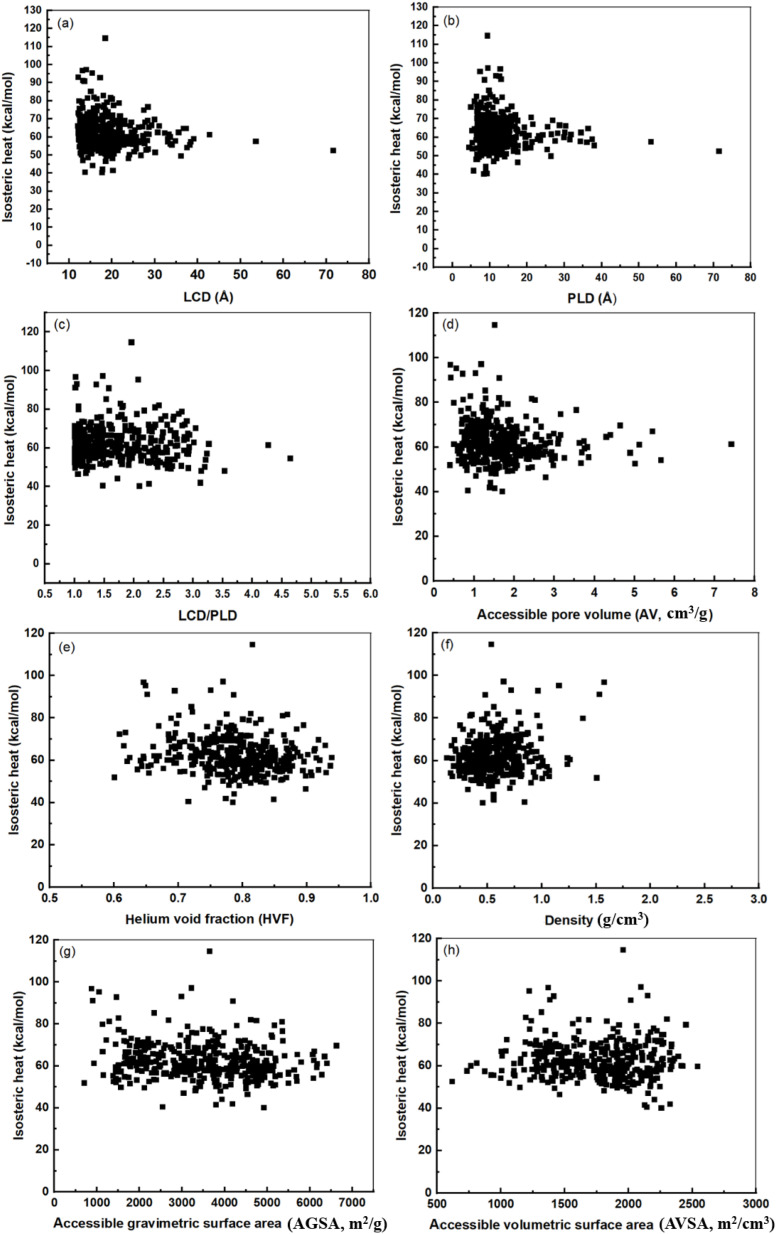
The correlation between isosteric heat of IVM and (a) LCD, (b) PLD, (c) LCD/PLD, (d) AV, (e) HVF, (f) density, (g) AGSA and (h) AVSA.

For a successful adsorption process, the ideal adsorbent should exhibit both high loading capacity and selectivity. In drug delivery systems, the diffusion rate plays a crucial role in selecting the appropriate MOF, as drug release must be precisely controlled. Alongside PLD, isosteric heat significantly influences the drug release profile. Furthermore, Fig. 2 illustrates the correlation between the isosteric heat of IVM and the structural properties of the 584 MOFs. This figure indicates no clear correlation between the structural characteristics of the MOFs (LCD, PLD, AV, HVF, density, AGSA, and AVSA) and the isosteric heats of IVM. The isosteric heats of the 385 adsorbing MOFs are relatively high, ranging from 114.56 kcal mol^−1^ to 40.11 kcal mol^−1^.

**Fig. 3:**
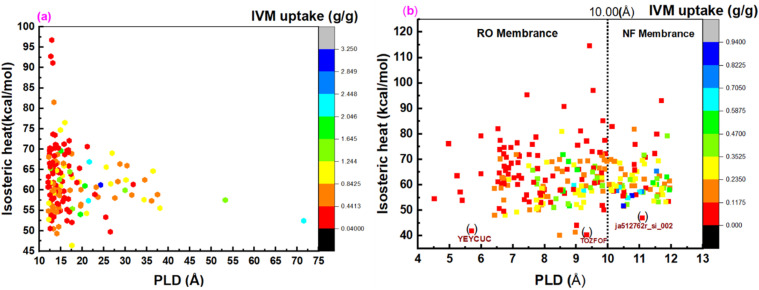
(a) The isosteric heat and loading capacity of (a) the 147 adsorbing MOFs with PLD > 12.0 Å; and (b) the 238 adsorbing MOFs with PLD <12.0 Å.

Isosteric heat refers to the energy released when a specific amount of an adsorbate is absorbed. It plays a crucial role in designing drug delivery systems, as it directly influences the drug release profile by determining the strength of interactions between the drug molecules and the MOF carrier. The optimal isosteric heat value varies depending on the drug and its intended release characteristics. A high isosteric heat value signifies strong drug–carrier interactions, making it suitable for gradual and controlled drug release. Conversely, a low isosteric heat value indicates weaker interactions, which facilitate faster drug release. Fig. 3a refers to the correlation between the PLD of 147 adsorbing MOFs and their isosteric heat and loading capacity. These MOFs have a PLD greater than 12.0 Å, which is larger than the IVM molecular diameter > 12.0 Å *d*^IVM^_diameter_. The isosteric heat values for the top five MOFs with the highest adsorption capacities—BAZGAM, XAHQAA, UHUPOD01, RAVXOD, and XAHPUT—are 61.24, 66.9, 57.37, 52.45, and 61.02 kcal mol^−1^, respectively. The overall isosteric heat values range from 46.4 kcal mol^−1^ (BIHBAX) to 96.73 kcal mol^−1^ (NALYEG). The average IVM uptake of NALYEG is 0.080 g g^−1^, whereas BIHBAX exhibits a significantly higher uptake of 0.906 g g^−1^, making it a highly suitable candidate for controlled drug release.

**Fig. 4:**
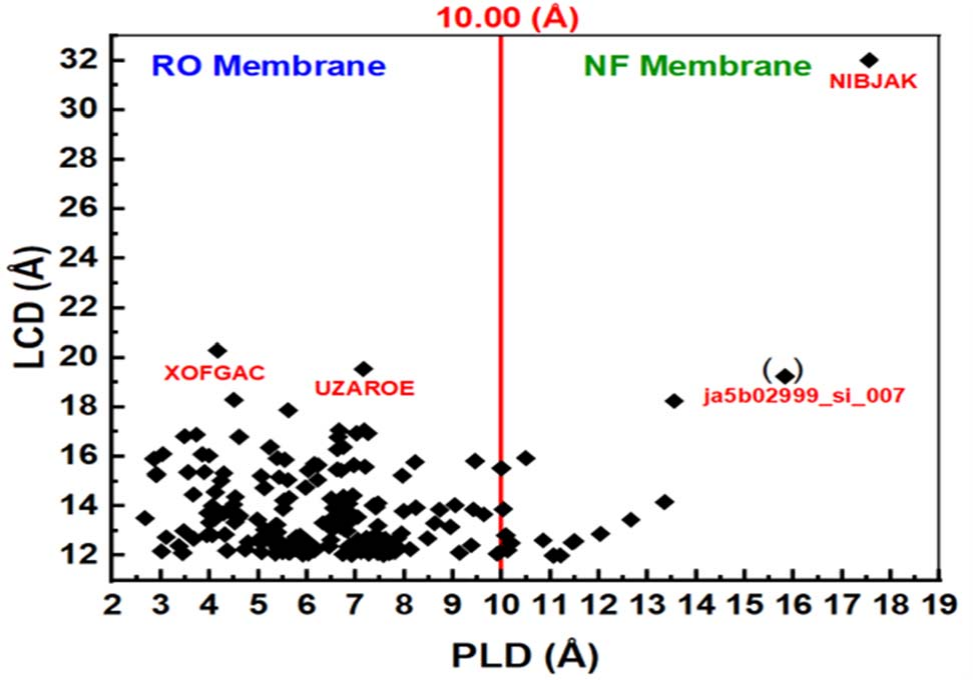
The LCD and PLD of the 199 non-absorbing MOF.

Fig. 3b shows the isosteric heat and loading capacity of the PLD of 238 adsorbing MOFs with PLD < 12.0 Å. The isosteric heat values for these MOFs range from 40.11 to 114.56 kcal mol^−1^, while their PLD values vary between 4.51 and 11.95 Å. Since the PLD of these MOFs is less than 12.0 Å, they are well-suited for membrane-based IVM separation due to their ability to permeate water, making them promising candidates for filtration and purification applications. This suggests that MOFs with PLD values between 4.51 and 10 Å are suitable for reverse osmosis (RO) membranes, while those with PLD values between 10.00 and 11.95 Å are ideal for nanofiltration (NF) membranes. Membrane fouling occurs when pollutants (such as IVM) accumulate and obstruct membrane pores due to interactions with the membrane surface, ultimately reducing water flow. MOFs with low isosteric heat exhibit weak IVM-MOF surface interactions, minimizing IVM fouling on the membrane. Among the best MOFs for RO membranes are YEYCUC and TOZFOF, while ja512762r_si_002 is the most suitable for NF membranes. The low IVM uptake of these MOFs further enhances their effectiveness in filtration applications. Additionally, the water permeability of these 238 adsorbing MOFs remains relatively high due to their large LCD values, making them highly efficient for separation processes. RO and NF membranes can also be fabricated using the 199 non-IVM absorbing MOFs. As shown in Fig. 4, based on their PLD values, NIBJAK and ja5b02999_si_007 are suitable for NF membranes, while XOFGAC and UZAROE are ideal for RO membranes. These MOFs possess the highest LCD values among the 199 non-IVM absorbing MOFs, making them promising candidates for efficient water separation applications.

Table 2 lists the top eight MOFs based on their isosteric heat values, which range from 114.56 to 90.8 kcal mol^−1^. These MOFs exhibit very low IVM adsorption capacities, ranging from 0.0625 to 0.0979 g g^−1^. Among them, only three MOFs—NALYEG, KIYXUN, and GAGSUF—have PLD values greater than 12.0 Å, measuring 12.881 Å, 12.648 Å, and 13.091 Å, respectively, making them suitable candidates for IVM adsorption. The remaining five MOFs have PLD values below 12.0 Å. A high isosteric heat indicates strong interactions between the MOF and the adsorbate, which is particularly advantageous for trace removal applications. Although the absolute adsorption capacities are low under the current simulation conditions, the strong binding affinity suggests that these MOFs can effectively adsorb IVM even at very low concentrations, as commonly found in contaminated water, especially when these MOFs are synthesized at the nanoscale.

**Table 2:** The top 8 MOFs according to their isosteric heat of IVM adsorption

Rank	Ref. code	Isosteric heat (kcal mol^−1^)	Uptake (g g^−1^)	LCD	PLD	LCD/PLD	Metal
1	LIKDOA	114.56	0.0625	18.483	9.419	1.962	Cu
2	OKABAE	97.06	0.0479	14.074	9.534	1.476	Cu
3	NALYEG	96.73	0.0809	13.122	12.881	1.018	U
4	AQOLID	95.29	0.0286	15.461	7.448	2.075	Co
5	CORZIU	93.02	0.0255	12.155	11.697	1.039	In
6	KIYXUN	92.75	0.0433	17.290	12.648	1.367	Cu,Mn
7	GAGSUF	91.13	0.0546	13.275	13.091	1.014	Th
8	FAGQAI	90.8	0.0979	13.611	8.614	1.580	Cu

**Fig. 5:**
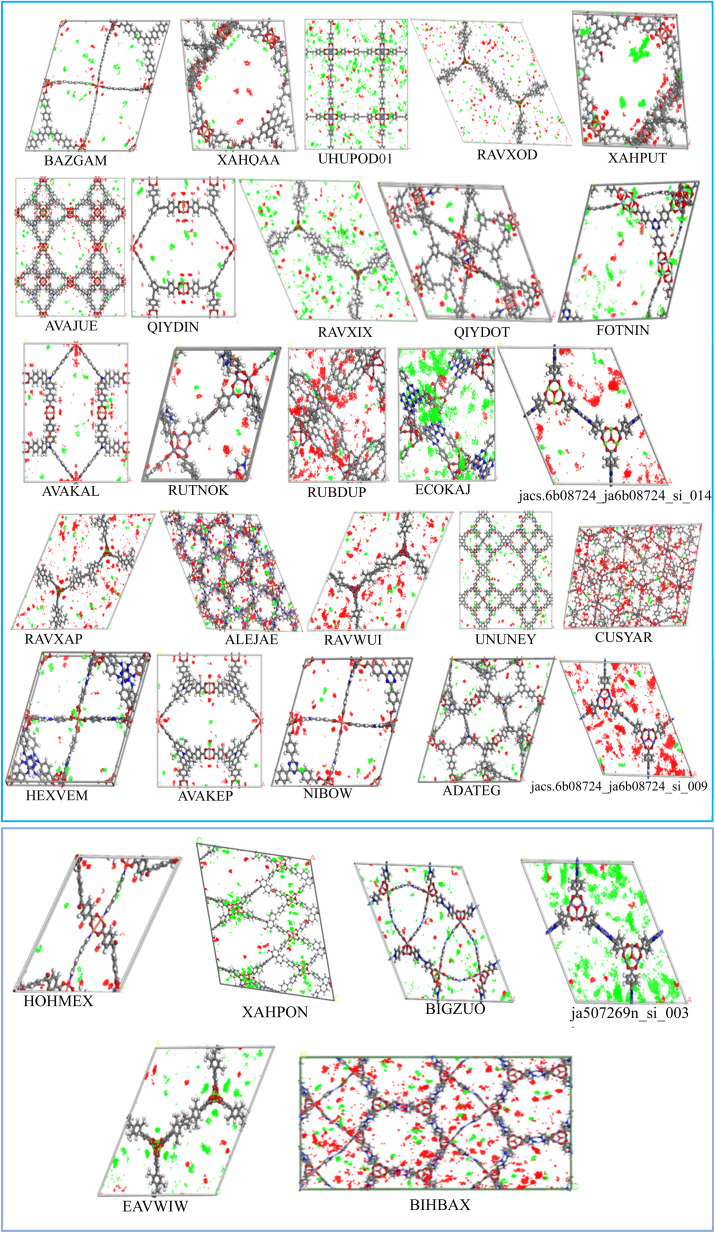
The density profile of adsorbed IVM in top 31 MOFs (water: red dots, ivermectin: green dots).

**Fig. 6:**
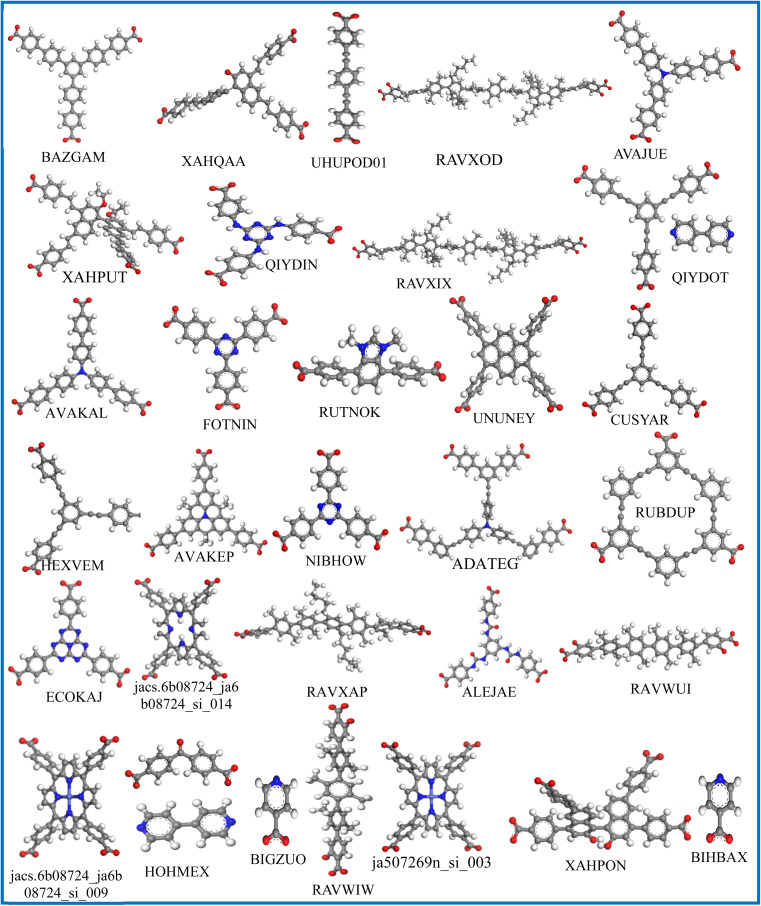
The structures of ligands used in the top 31 loading MOFs.

To evaluate the adsorption behavior, the density profiles for the absorbed water and IVM molecules in the top 31 MOFs at 298 K are shown in Fig. 5. Water molecules (red dots) predominantly surround polar functional groups and metal centers due to their hydrophilic nature, while IVM molecules (green dots) tend to accumulate in hydrophobic voids within the MOF structures. This behavior highlights the importance of structural features, such as hydrophobic domains, pore size, and surface chemistry, in determining the adsorption and spatial distribution of guest molecules. MOFs such as ECOKAJ, ja507269n_si_003, RAVXIX, and UHUPOD01 display significantly more green dots, indicating a higher density of adsorbed IVM molecules. This suggests the presence of multiple favorable interaction sites and a highly accessible porous environment for IVM diffusion and stabilization. These MOFs possess large cavities and well-balanced hydrophobic-hydrophilic domains that facilitate strong binding and high occupancy of IVM molecules. The increased density of green regions is therefore associated not only with higher loading capacities but also with enhanced affinity and retention of IVM, even at low concentrations, making them promising candidates for trace drug removal from water. Fig. 6 displays the structures of the ligands used in the synthesis of these top 31 MOFs. The MOFs that showed strong adsorption of IVM typically feature ligands with aromatic backbones and delocalized π-electron systems, which help maintain the structural integrity of the frameworks. Many of these ligands also include heteroatoms such as nitrogen and oxygen. The aromatic components enable π–π stacking with the aromatic parts of the IVM molecule, while the heteroatoms act as potential sites for hydrogen bonding. Such synergistic interactions between ligand functionalities and IVM molecules help explain the enhanced adsorption performance observed in these MOFs.

### 3.2. MD simulation

**Fig. 7:**
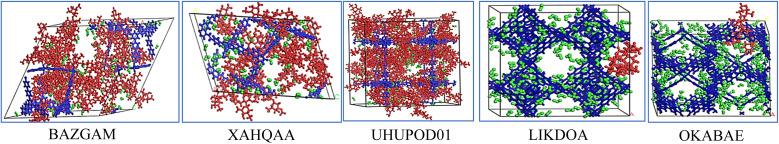
The final MD snapshots of ivermectin, water, and MOFs; red color for ivermectin, blue color for MOF, and green color for water.

To investigate the interactions between IVM, water, and MOFs, we performed MD simulation on the top three MOFs with the highest loading capacities—BZGAM, XAHQAA, and XAHPUT—as well as two MOFs with the highest isosteric heats, UHUPOD01 and AVAJUE. These simulations provide insights into the adsorption mechanisms, molecular interactions, and stability of the drug-MOF-water systems, helping to evaluate their suitability for controlled drug delivery and separation applications. Fig. 7 presents the final MD snapshots of these systems, illustrating the spatial distribution of IVM and water molecules within the MOF frameworks.

**Fig. 8:**
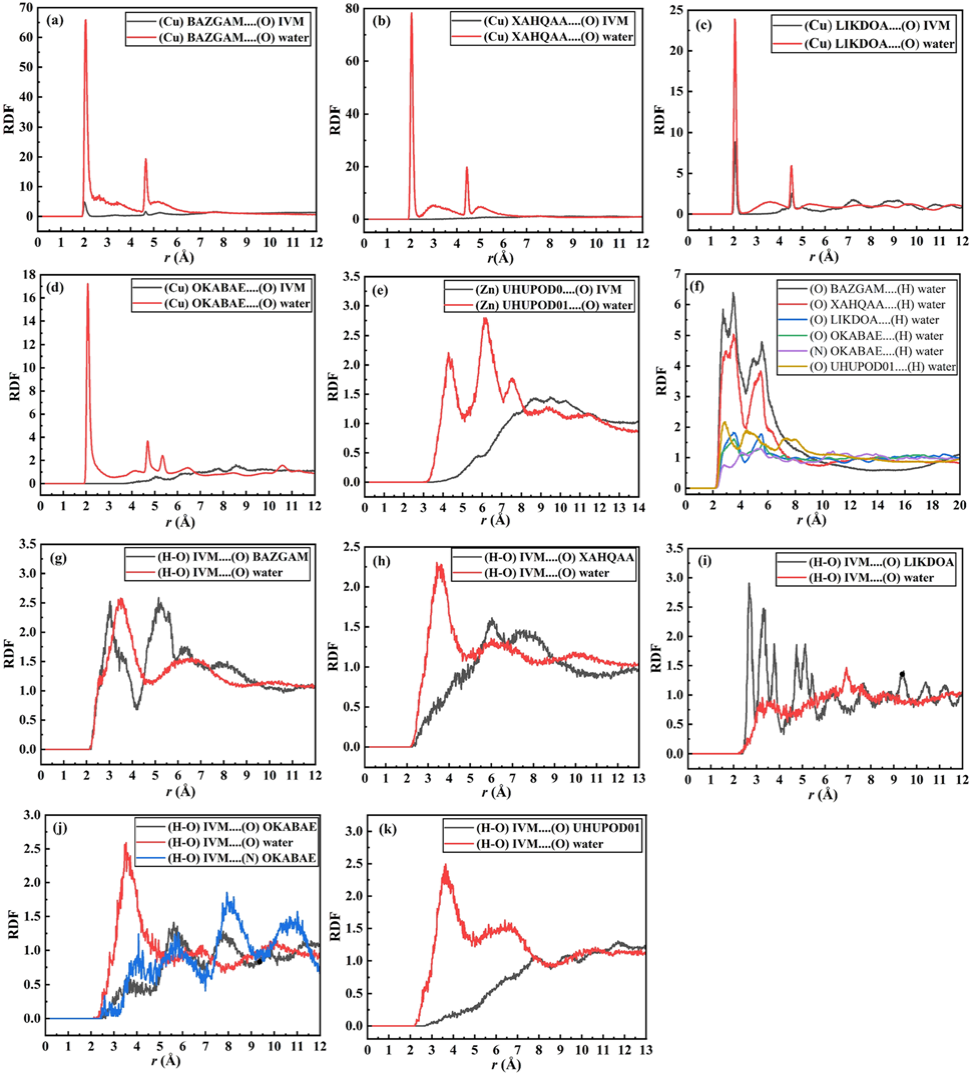
The RDF analysis results from the MD simulation of IVM and water in BAZGAM, XAHQAA, LIKDOA, OKABAE and UHUPOD01; (a–e) RFDs of (Cu or Zn)MOFs⋯(O)IVM/water interactions; (f) RDFs of (O/N)MOFs…(H)water interactions; (g–k) RDFs of (H–O)IVM⋯(O/N)MOFs and (O)water interactions.

Based on the simulation trajectories, the radial distribution function (RDF) was computed to evaluate the interactions between ivermectin, MOFs, and water molecules. The RDF is an important tool used to detect the possibility of finding any particle through the radial distances (*r* + d*r*) from the origin element. The type of adsorption, bond length, and bond nature are all ascertained using the RDF.^56^Fig. 8a–e shows the interaction between the metal of the MOFs and the oxygen (O) atoms of the IVM, (Cu/Zn)MOF⋯(O)IVM. The peak of the interaction of BAZGAM, LIKDOA, and OKABAE appeared at a distance of ∼2.03 Å, 2.07 Å, 5.03 Å with a minor peak, respectively. There was no interaction between (Cu)XAHQAA and (O) atoms of IVM. The peak of (Zn)UHUPOD01⋯(O)IVM appeared at a distance of ∼5.71 Å. This finding indicates that there is high affinity between the IVM's oxygen atom and the Cu metal of BAZGAM and LIKDOA, and a low affinity toward the Cu metal of OKABAE. The interaction of (O) atoms of IVM with (Cu) atom is higher than in the case of (Zn) metal. Fig. 8a–e also illustrates the interactions between water's oxygens (O) and Cu/Zn of MOFs, (Cu/Zn)MOF⋯(O)water. The peak of the interaction of BAZGAM, XAHQAA, LIKDOA, and OKABAE appeared at a distance of ∼2.05 Å, 2.05 Å, 2.07 Å and 2.07 Å with a high peak, respectively. The peak of (Zn)UHUPOD01⋯(O)water of appeared at a distance of ∼4.29 Å. This finding indicates that is high affinity between the oxygen atom of the water and the (Cu) atoms of MOF more than the (Zn) atoms of UHUPOD01. Fig. 8f illustrates the formation of hydrogen bonds between the polar atoms of the MOFs (O and N) and the hydrogen atoms of water molecules. The RDF results for the (O)MOF⋯(H)water interactions reveal that BAZGAM and XAHQAA exhibit the same peak at approximately 2.73 Å, while OKABEA and LIKDOA share a peak at ∼3.51 Å. Additionally, UHUPOD01 shows a peak at ∼2.83 Å. The water's hydrogen atoms interacts with (N) atom of OKABEA at a distance of ∼2.79 Å. These findings confirm the presence of water molecules surrounding the metal centers and polar atoms of the MOFs, highlighting the role of hydrogen bonding in water adsorption and molecular interactions within the MOF structures. The intermolecular interaction of IVM with MOFs and water is primarily observed through hydrogen bonding, where the hydrogen atom from the hydroxyl group (HO) of IVM forms hydrogen bonds with the oxygen atoms of both the MOFs and water molecules. Fig. 8g–k presents the RDF results for the interactions between the (H–O)IVM and the oxygen atoms of various MOFs. The RDF peaks occur at approximately 3.03 Å for BAZGAM, 6.03 Å for XAHQAA, 2.67 Å for LIKDOA, 7.83 Å for UHUPOD01, and 3.63 Å for OKABEA. Among these, the interaction strength between IVM and BAZGAM, as well as LIKDOA, is higher compared to the other MOFs. The RDF peak of (H–O)IVM⋯(N)OKABEA at ∼2.59 Å, which is a very minor peak. The hydroxyl's hydrogen of IVM also formed hydrogen bonds with the (O)water (Fig. 8g–k). RDF shows a peak of (H–O)IVM⋯(O)water in BAZGAM, XAHQAA, LIKDOA, OKABEA and UHUPOD01 at distance of ∼3.49 Å, 3.41 Å, 3.25 Å, 3.53 Å, and 3.65 Å, respectively. The interaction behavior of water with IVM in all MOFs is quite similar, with simple differences.

### 3.3. Water-stable MOFs

Water can compromise the structural integrity of many MOFs by attacking metal–ligand coordination bonds, particularly those involving hydrolysis-sensitive metal nodes or weak linker interactions.^57^ This challenge is particularly critical in water treatment applications, where the adsorbent is continuously exposed to aqueous environments. Therefore, identifying water-stable MOFs is essential to ensure the practical viability of high-performing materials for IVM adsorption. Recent computational advancements, such as MOFSimplify,^57^ offer valuable tools to predict MOF water stability. MOFSimplify is a machine learning-based platform trained on curated experimental data using graph-based structural representations. It employs a binary classification model to categorize MOFs as water-stable (including highly stable “HK” and thermodynamically stable “TS” classes) or water-unstable (including unstable “U” and low kinetic stability “LK” classes). The classification relies on features such as metal–ligand bonding motifs, pore geometry, and revised autocorrelation (RAC) fingerprints. As shown in Table 3, out of 117 MOFs with PLD > 12.00 Å and TCS uptake higher than commercial charcoal, 59 were classified as water-stable. UHUPOD01, QIYDOT, FOTNIN, and UNUNEY demonstrated exceptional uptake (>1.5 g g^−1^) while maintaining structural integrity, highlighting their potential for real-world water purification. It is worth mentioning here that among the 8 top MOFs according to their isosteric heats of IVM adsorption (Table 3), LIKDOA, AQOLID, CORZIU, and AGQA were found to be water-stable MOFs. It is also worth noting that among the eight MOFs with the highest isosteric heats of IVM adsorption (Table 3), LIKDOA, AQOLID, CORZIU, and AGQA were predicted to be water-stable, reinforcing their potential for both strong binding and operational durability.

**Table 3:** Water-stable MOFs identified among the 117 TCS-adsorbing MOFs with PLD > 12.00 Å, as predicted by the MOFSimplify machine learning model

Rank	Ref. code	Uptake (g g^−1^)	Water stability		Rank	Ref. code	Uptak (g g^−1^)	Water stability	
			Confidence score					Confidence score	
1	UHUPOD01	2.109	0.87	Stable	31	ja408959g_si_002	0.525	0.99	Stable
2	QIYDOT	1.562	0.56	Stable	32	BEDYEQ	0.482	0.88	Stable
3	FOTNIN	1.535	0.87	Stable	33	QEFWUV	0.455	0.89	Stable
4	UNUNEY	1.495	0.59	Stable	34	ZARLEL	0.451	0.93	Stable
5	RUBDUP	1.166	0.76	Stable	35	XUTQEI	0.433	0.47	Stable
6	ECOKAJ	1.150	0.81	Stable	36	UBULIO	0.411	0.51	Stable
7	ALEJAE	1.072	0.81	Stable	37	HUYKIV	0.409	0.68	Stable
8	RAVWUI	1.051	0.55	Stable	38	BUXZAX	0.404	0.91	Stable
9	jacs.6b08724_ ja6b08724_si_009	1.018	0.57	Stable	39	MALROJ	0.345	0.52	Stable
10	HOHMEX	0.942	0.61	Stable	40	DOMDAL	0.337	0.75	Stable
11	ja507269n_si_003	0.928	0.58	Stable	41	MALRUP	0.332	0.63	Stable
12	ja512973b_si_004	0.885	0.7	Stable	42	IZERAI	0.330	0.66	Stable
13	jacs.6b01663_ ja6b01663_si_003	0.827	0.88	Stable	43	DADLOM	0.322	0.79	Stable
14	PIBNUK01	0.814	0.84	Stable	44	XIGFOJ	0.322	0.66	Stable
15	PIBNUK	0.803	0.84	Stable	45	ja5b02999_si_004	0.321	0.68	Stable
16	YODWOF	0.800	0.59	Stable	46	XOXMED	0.306	0.67	Stable
17	TOVKOG	0.794	0.55	Stable	47	ja300034j_si_002	0.291	0.54	Stable
18	PIBPIA	0.721	0.74	Stable	48	LETQEI	0.281	0.7	Stable
19	ja507269n_si_002	0.694	0.56	Stable	49	YOZQEK	0.280	0.69	Stable
20	BUNLAZ	0.691	0.89	Stable	50	ja5b02999_si_002	0.267	0.74	Stable
21	XAFFAN	0.675	0.76	Stable	51	ATIJUJ	0.246	0.69	Stable
22	EMIZAD	0.608	0.93	Stable	52	OJIDAN	0.241	0.57	Stable
23	XAFFER	0.607	0.76	Stable	53	RUYVIS	0.239	0.98	Stable
24	VAGMEX	0.607	0.76	Stable	54	WOLYON	0.232	0.93	Stable
25	KAWHEY	0.564	0.75	Stable	55	FUNCEX	0.227	0.94	Stable
26	ALULEZ	0.560	0.61	Stable	56	NUTYEI	0.224	0.82	Stable
27	VAGMAT	0.558	0.76	Stable	57	XIDSUZ	0.224	0.77	Stable
28	ADATIK	0.554	0.53	Stable	58	ABEXEN	0.218	0.71	Stable
29	FIFGEI	0.541	0.63	Stable	59	NUTYIM	0.210	0.76	Stable
30	ja4050828	0.534	0.9	Stable					

## 4. Conclusion

Computational screening has proven to be a powerful and efficient technique for identifying the best MOF candidates for IVM removal from water. In this study, over 14 000 porous three-dimensional MOF structures were evaluated, and 584 MOFs were selected for further simulations. MC simulations were employed to determine IVM adsorption, followed by MD simulations on five selected systems to gain deeper insights into adsorption behavior. Several factors influence the loading capacity and isosteric heat of MOFs with IVM, including PLD, LCD, the LCD/PLD ratio, AV, HVF, AGSA, and AVSA. Based on their PLD values and loading capacities, MOFs were categorized into three functional groups: adsorption, membrane separation, and drug delivery. Out of the 584 MOFs, 147 exhibited significant IVM adsorption, making them suitable for IVM adsorbents and drug delivery applications. The remaining 437 MOFs were more effective for membrane filtration, either as reverse osmosis or nanofiltration membranes. The top 15 MOFs identified for IVM adsorption and drug delivery are BAZGAM, XAHQAA, UHUPOD01, RAVXOD, XAHPUT, AVAJUE, QIYDIN, RAVXIX, QIYDOT, FOTNIN, AVAKAL, RUTNOK, UNUNEY, CUSYAR, and HEXVEM, with loading capacities ranging from 1.205 to 3.248 g g^−1^. Among these high-performing candidates, UHUPOD01, QIYDOT, FOTNIN, and UNUNEY were predicted to be water-stable, reinforcing their potential for real-world application in aqueous drug delivery and water treatment environments. For membrane filtration applications, NIBJAK and ja5b02999_si_007 were the best candidates for NF membranes, while XOFGAC and UZAROE were optimal for RO membranes. Notably, copper-based MOFs dominated the top-performing structures. Density profile analyses revealed that IVM molecules predominantly accumulate in hydrophobic voids, while water molecules interact with the polar sites and metal centers within the MOF structures. These findings provide a strong foundation for designing and optimizing MOFs for effective IVM removal, drug delivery, and membrane filtration applications.

## Data availability

The data supporting the findings of this study, including the computational screening results, Grand Canonical Monte Carlo (GCMC) simulation outputs, and molecular dynamics simulation data, are available from the corresponding author upon reasonable request. Any additional datasets or analysis used in this study can also be provided upon request.

## Conflicts of interest

The authors declare that they have no known competing financial interests or personal relationships that could have appeared to influence the work reported in this paper.

## Supplementary Material

RA-015-D5RA01662B-s001
